# Assessment of In Vitro Digestive Behavior of Lactic-Acid-Bacteria Fermented Soy Proteins: A Study Comparing Colloidal Solutions and Curds

**DOI:** 10.3390/molecules27217652

**Published:** 2022-11-07

**Authors:** Yaqiong Wang, Yumeng Fu, Elham Azarpazhooh, Wei Li, Qi Liu, Xin Rui

**Affiliations:** 1 College of Food Science and Technology, Nanjing Agricultural University, Nanjing 210095, China; 2 Department of Agricultural Engineering Institute, Khorasan Razavi Agricultural and Natural Resources Research and Education Center, AREEO, Mashhad 1696700, Iran; 3 Department of Information Engineering, Nanjing Institute of Mechatronic Technology, Nanjing 211306, China

**Keywords:** soy protein, lactic acid bacteria, in vitro dynamic gastrointestinal digestion, digestomics

## Abstract

This study investigated the effect of lactic-acid-bacteria fermentation on the microstructure and gastrointestinal digestibility of soy proteins using a digestomics approach. Fermented soy protein isolates (FSPIs) under varied fermentation-terminal pH demonstrated a colloidal solution (FSPI-7.0/6.0) or yogurt-like curd (FSPI-5.0/4.0) state. Cryo-electron microscopy figures demonstrated the loosely stacked layer of FSPI-7.0/6.0 samples, whereas a denser gel network was observed for FSPI-5.0/4.0 samples. Molecular interactions shifted from dominant ionic bonds to hydrophobic forces and disulfide bonds. The gastric/intestinal digestion demonstrated that the curd samples afforded a significantly low particle size and high-soluble protein and peptide contents in the medium and late digestive phases. A peptidomics study showed that the FSPI-6.0 digestate at early intestinal digestion had a high peptidome abundance, whereas FSPI curd digestates (FSPI-5.0/4.0) elicited a postponed but more extensive promotion during medium and late digestion. Glycinin G2/G4 and β-conglycinin α/α’ subunits were the major subunits promoted by FSPI-curds. The spatial structures of glycinin G2 and β-conglycinin α subunits demonstrated variations located in seven regions. Glycinin G2 region 6 (A349–K356) and β-conglycinin α subunit region 7 (E556–E575), which were located at the interior of the 3D structure, were the key regions contributing to discrepancies at the late stage.

## 1. Introduction

Soy emulsion has attracted increasing attention due to its nutritional value and desirable processing properties, especially for consumers with lactose intolerance and cow-milk allergy [1]. Soy emulsion is a colloidal solution obtained using water’s extraction of soybeans. It has been recommended by the United States Food and Drug Administration as a healthy food [2]. In addition, the consumption of soy emulsion provides several health benefits, including alleviating the risks of cancer and cardiovascular and neurodegenerative diseases [3].

Fermented soy emulsion is obtained by fermenting soy emulsion by lactic acid bacteria (LAB), a process that results in a set-type yogurt-like product. LAB fermentation of soy emulsion results in the acid gelation of soy protein [4]. Given the important influence of acid gelation on the textural properties of fermented soy emulsion, the gelation capability of soy protein isolates (SPIs) has been extensively studied [5,6]. Studies on gelation mechanism demonstrated that LAB growth in soy emulsion results in a gradual release of protons and in a decrease in pH, allowing soy proteins to coagulate and form a yogurt-like curd [7]. Acid gelation by LAB caused a relatively slow acidification and led to the formation of a gel network with large pores and thick strands, which was different from the curd generated by an acidic coagulant [8]. Studies demonstrated that LAB fermentation caused the alteration in SPI particle size and microstructure [5]. This prior research provided a solid knowledge base with respect to the physiochemical and structural characteristics of LAB-induced soy protein curds.

The varied structural characteristics of a particular food could lead to differences in the release of macronutrient components from a complex food matrix to the intestinal lumen [9]. The gelation of β-casein and egg white proteins change and postpone the digestive progress in pepsin digestion, due to the steric hindrance of large macromolecules through the network pores [10,11]. However, a limited number of studies unveiled the relationship between LAB-induced soy protein gelation and digestive behavior. This gap caught the attention of researchers, given the restricted digestibility of soy proteins [12], which is partially related to the high resistance to digestive proteases mediated by the glycinin basic subunit and the β-conglycinin α’ and α subunits [13,14].

Digestomics has become a valuable tool in studying the protein composition of food gastrointestinal digestates, including the identification and quantitative mapping of peptides [15]. Thus, LAB-fermented SPIs (FSPIs) were prepared in this study. The interactions between proteins and structural properties were investigated. In addition, FSPIs with varied structural features were subjected to a dynamic gastrointestinal model (Bionic Rat Model II+), and the protein molecular degradation behavior was investigated by peptidomics. This study provides fundamental information for understanding the acid gelation of soy-based yogurt and protein gastrointestinal digestive behaviors.

## 2. Results

### 2.1. Growth of Lactobacillus Plantarum B1-6 and SPI Acidification

**Figure 1:**
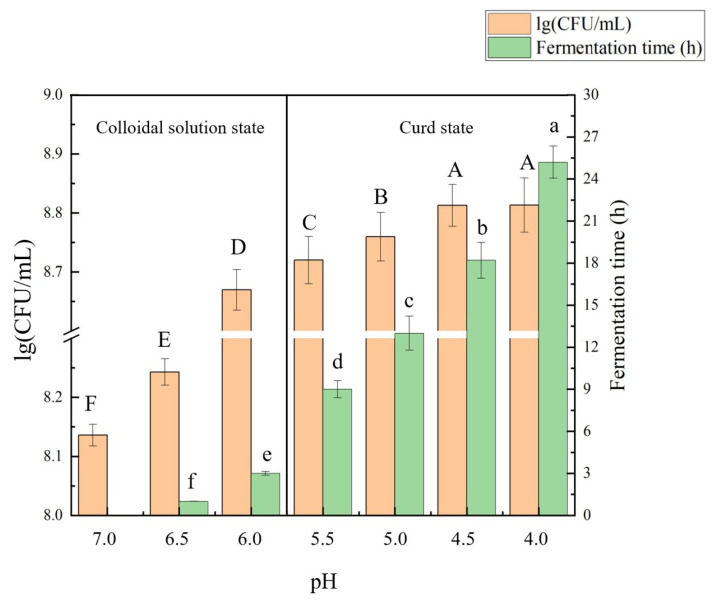
Fermentation time, viable cell counts, and interaction between proteins of FSPI at varied levels of terminal pH. Different letters indicated significant difference (*p* < 0.05) between the FSPIs at different levels of terminal pH.

The SPIs had a starting pH of 7.0. The growth of *L. plantarum* B1-6 in SPI allowed a gradual decline in pH, which might have led to protein gelation under a certain pH value [16]. Figure 1 shows the fermentation time and viable LAB cell counts at varied terminal pH. The logarithmic growth period of *L. plantarum* B1-6 was 0 h to 18 h, and a significant decline in pH was observed during this period (pH 7.0–4.5). At 0–3 h, *L. plantarum* B1-6 elicited a rapid growth, accompanied by a fast drop in pH, from 7.0 to 6.0. The pH drop rate decreased thereafter, which was probably due to the occurrence of acid gelation. At pH 5.5, the soy protein colloidal solution was converted to soy protein curd, suggesting that the onset gelation pH was between 6.0 and 5.5. This finding was in accordance with that of a previous study [17]. The logarithmic growth of *L. plantarum* B1-6 was terminated at 18 h, when the pH reached 4.5 and progressed to a stationary phase. Although viable LAB cell counts demonstrated no significant change at this stage, the pH of the SPIs slowly dropped from 4.5 to 4.0. According to these results, four pH levels (pH 7.0, 6.0, 5.0, and 4.0) representing varied growth periods of *L. plantarum* B1-6 and different physiochemical states (colloidal solution/curd) were selected to perform further studies. They were abbreviated as fermented SPI (FSPI)-7.0, FSPI-6.0, FSPI-5.0, and FSPI-4.0. FSPI-7.0 and FSPI-6.0 were in a colloidal solution state, whereas FSPI-5.0 and FSPI-4.0 were in a curd state.

### 2.2. Interactions between Soy Proteins

**Figure 2:**
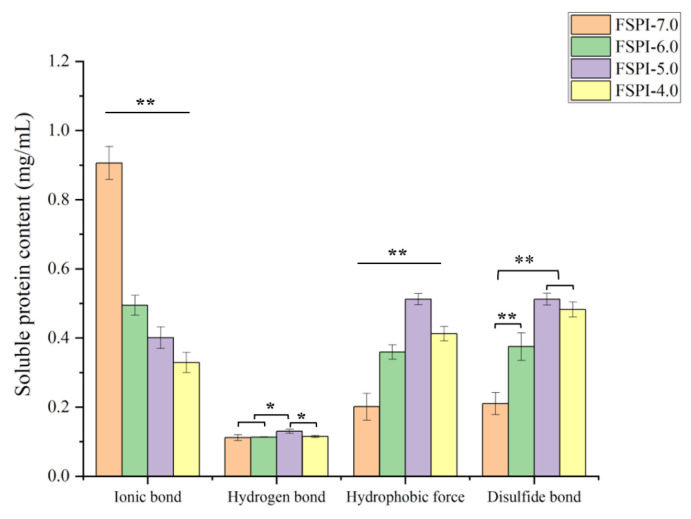
Interaction (ionic bond, hydrogen bond, and hydrophobic forces) between proteins of FSPI at varied levels of terminal pH. Analysis of variance level, * *p* < 0.05, ** *p* < 0.01.

Protein gelation forms a 3D network stabilized by non-covalent and covalent forces [18]. Figure 2 shows the proportions of soy proteins stabilized by non-covalent forces (ionic bond, hydrogen bond, and hydrophobic force) and a covalent force (disulfide bond). FSPI-7.0 was mainly maintained by ionic bonds, followed by hydrophobic interactions, disulfide bonds, and hydrogen bonds in descending order. Ionic-bond interaction was the predominant interaction in FSPI-6.0, but the proportion significantly decreased. Further fermentation (FSPI-5.0 and FSPI-4.0) allowed a major shift of the dominant interactions from ionic bonds to hydrophobic forces and disulfide bonds, suggesting that the two interactions were the major forces that maintained the gel network. The result was consistent with that of a previous study [19]. This finding may also be related to the decrease in electrostatic repulsion and net charge as the pH approached the isoelectric point of soy proteins [20].

### 2.3. Microstructure of FSPI Samples

**Figure 3:**
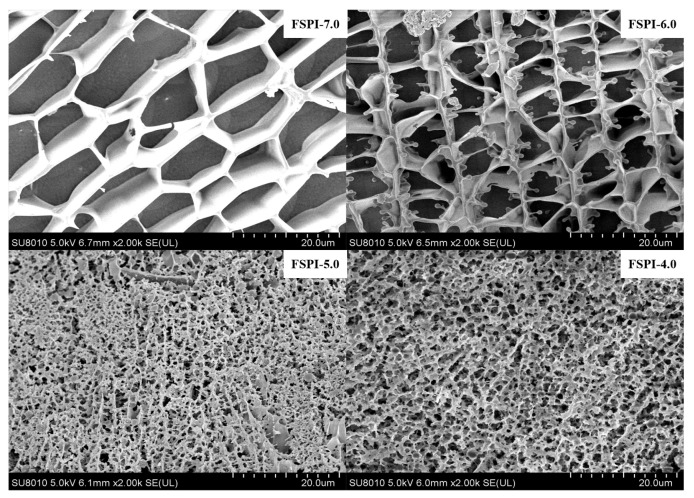
Cryo-EM microstructure of FSPIs at varied levels of terminal pH.

The microstructure of FSPIs was observed by cryo-EM (Figure 3). FSPI-7.0 presented a typical protein network composed of loose sheets with thick strands and large, uneven, rectangle hollows with a diameter of approximately 18 µm (Figure 3. FSPI-6.0 showed a similar microstructure with FSPI-7.0, but showed smaller hollows at a diameter of approximately 7 µm (Figure 3. The microstructure formed in colloidal solution matrices (FSPI-7.0 and FSPI-6.0) may be related to the filamentous nature of soy protein [19]. FSPI-5.0 (Figure 3 and FSPI-4.0 (Figure 3 had a 3D and dense gel network, with pores of a diameter of approximately 1.8 µm, in which FSPI-4.0 demonstrated a more uniform network arrangement. This finding may be related to a previous result that showed the increment in hydrophobic force and disulfide interactions between protein particles. A similar structure was found in acid-induced soymilk, which was a 3D stranded gel network with a crosslink among protein polymers [21].

### 2.4. Dynamic Gastrointestinal Digestion of SPI and FSPIs

#### 2.4.1. Gastric Digestion

**Figure 4:**
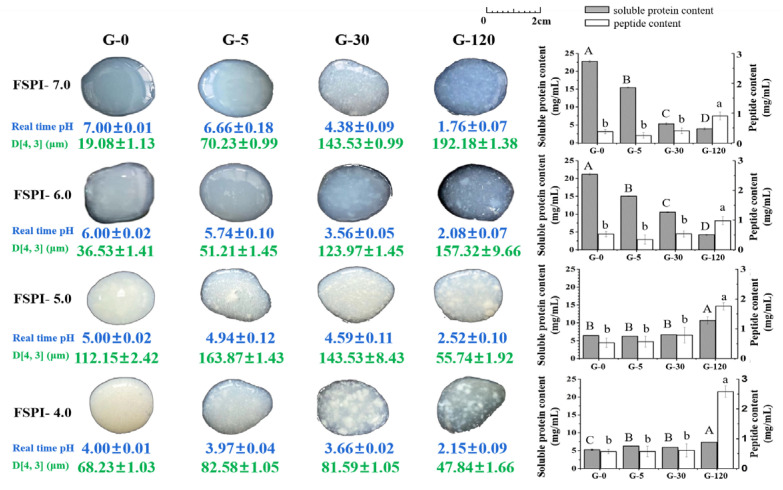
Intuitive images, D [4,3] value, real time pH, soluble protein, and peptide content of FSPI digestates collected during dynamic gastric digestion. G-5, G-30, and G-120 represent gastric digestion at 5, 30, and 120 min, respectively. Different letters indicated significant difference (*p* < 0.05) between the FSPIs at different gastric digestion stages. Time is denoted by a star above the bars.

Figure 4 shows the intuitive image of FSPI gastric digestates and their physiochemical characteristics—apparent real time pH, particle size (D [4,3]), soluble protein, and peptide content—during 120 min of gastric digestion.

##### Protein Particle Size

At the initial stage of gastric digestion (G-0), FSPIs at varied fermentation-terminal pH showed D [4,3] values of 19.08 ± 1.13 (FSPI-7.0), 36.53 ± 1.41 (FSPI-6.0), 112.15 ± 2.42 (FSPI-5.0), and 68.23 ± 1.03 µm (FSPI-4.0). The evolvement of greater particles during fermentation was probably related to the loss of ionic interactions and an increment in the hydrophobic and disulfide bonds, leading to a compact gel network. This finding was supported by that of a previous study [21]. FSPI-4.0 demonstrated relatively smaller particles compared with FSPI-5.0, indicating the partial depolymerization of protein particles.

Colloidal solution digestates (FSPI-7.0 and FSPI-6.0) collected at G-5 showed images similar to those of G-0, but demonstrated a great improvement in their D [4,3] values (51.21–70.23 µm). The particles were continuously enlarged along with gastric digestion and peaked at G-120, showing a D [4,3] value 4.3–10.1-fold (*p* < 0.05) higher than that of the digestates at G-0. Images of the digestates collected at G-120 showed coarse and uneven particles, which might have contributed to the formation of large aggregates. The changes in the D [4,3]-value of the gastric digestates from curd samples (FSPI-5.0 and FSPI-4.0) differed from those of the colloidal solution digestates. The curd digestates (FSPI-5.0 and FSPI-4.0) showed a slight increment in D [4,3] value (82.58–163.87 µm) at G-5, but the particle size sharply decreased along with gastric digestion and reached the lowest value at G-120 (47.84–55.74 µm). The value was significantly lower than that of the corresponding colloidal solution digestates at G-120 (157.32–192.18 µm)

The particle size of gastric digestates was closely related to real-time apparent pH. For those pH values obtained at early and medium gastric digestion times (G-5 and G-30, respectively) of the curd digestates and the medium gastric digestion time (G-30) of the colloidal solution digestates, the real-time apparent pH values were 3.97–4.94, which were close to the isoelectric point of soy protein (at approximately 4.5). However, although the hydrocolloid/curd digestates obtained at G-120 demonstrated similar real-time apparent pH, the particle sizes of the two groups samples at G-120 significantly differed. The aggregates were observed in the figure of the curd digestates at G-120 (Figure 4), and their particle size was small. This finding indicated that acidic aggregates formed in the curd digestates during late gastric digestion, but their structure was fragile and dissociated rapidly after the dispersion procedure of ILLS. This feature was not found in the colloidal solution digestates.

##### Soluble Protein Content

Soluble protein content is an index that indicates bio-accessible proteins that are ready for proteolysis [22]. FSPI-7.0 showed the highest soluble protein content (22.77 ± 0.28 mg/mL) at the initial stage of gastric digestion, followed by FSPI-6.0 (21.20 ± 0.27 mg/mL), whereas significantly lower values were obtained for FSPI-5.0 (6.41 ± 0.03 mg/mL) and FSPI-4.0 (5.25 ± 0.21 mg/mL). This finding indicated that soy protein lost its solubility due to the formation of protein curds. A similar result was observed in our previous study [23]. At the early and medium stages of gastric digestion (0 min to 30 min), the FSPI-7.0 digestate demonstrated a significant reduction in soluble protein content (5.32 ± 0.26 mg/mL), whereas the FSPI-6.0 digestate maintained a higher soluble protein content (10.55 ± 0.14 mg/mL). The curd digestates (FSPI-5.0 and FSPI-4.0) showed a slight increase (5.91–6.64 mg/mL) (G-30), although the value was lower than that of FSPI-6.0. Subsequently, the FSPI-5.0 digestate at G-120 (10.67 ± 1.05 mg/mL) afforded a significant climb in soluble protein content, followed by FSPI-4.0 (7.33 ± 0.04 mg/mL), whereas the colloidal solution digestates showed a rapid decrease at G-120 with values of 3.91 ± 0.27 (FSPI-7.0) and 4.22 ± 0.16 mg/mL (FSPI-6.0). The result indicated that the colloidal solution samples exerted a fast gastric-emptying progress, compared with the curd samples. The dynamic physiochemical changes in the gastric digestion caused the soy proteins that were originally trapped in curd matrices to be released and to become soluble during late gastric digestion. This result was in accordance with that of a previous study, in which dairy-acidified curd possessed higher soluble protein content during late gastric digestion, compared with dairy milk [22].

##### Peptide Content

FSPI-4.0 showed the highest peptide content (0.57 mg/mL) followed by FSPI-5.0 (0.54 mg/mL) and FSPI-6.0 (0.53 mg/mL), whereas a drastically lower value (0.37 mg/mL) was obtained for FSPI-7.0. This result demonstrated the potential proteolytic hydrolysis capacity of *L. plantarum* B1-6, which was supported by a previous study [24]. A negligible increment in peptide content was observed at early and medium gastric digestion stages (0min to 30 min) for all investigated samples, whereas a pronounced increment was observed between G-30 and G-120, especially in the curd digestates. The FSPI-5.0 and FSPI-4.0 digestates collected at G-120 showed 3.3–4.5-fold improvement, with values of 1.76 ± 0.32 and 2.58 ± 0.30 mg/mL, respectively, compared with FSPI-7.0 and FSPI-6.0. This result suggested the greater capacity of the FSPI curd samples (FSPI-5.0 and FSPI-4.0) to release peptides in late gastric digestion, compared with the colloidal solution samples (FSPI-7.0 and FSPI-6.0). The formation of curd matrices may lead to enhanced enzymatic susceptibility of soy proteins and increased chances of peptide liberation [25].

#### 2.4.2. Intestinal Digestion

**Figure 5:**
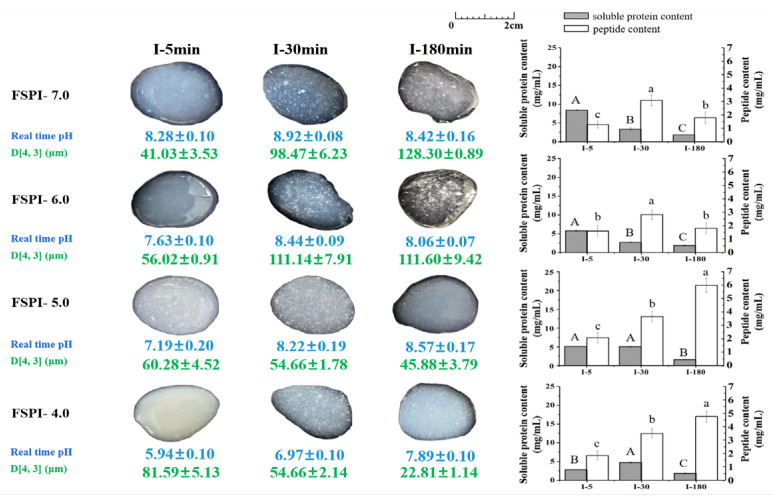
Intuitive images, D [4,3] value, real time pH, soluble protein, and peptide content of FSPI digestates collected during dynamic intestinal digestion. I-5, I-30, and I-180 denote intestinal digestion at 5 min, 30 min, and 180 min, respectively. Different letters indicate significant difference (*p* < 0.05) between the FSPIs at different intestinal digestion stages.

Figure 5 shows the intuitive image and physiochemical characteristics of the FSPI intestinal digestates—apparent real time pH, particle size (D [4,3]), soluble protein, and peptide content—during 180 min of digestion.

##### Protein Particle Size

The FSPI-7.0 and FSPI-6.0 digestates at I-5 showed a uniform colloidal solution with a lower D [4,3] value (41.03–56.02 µm), compared with those of the curd samples (60.28–81.59 µm). However, in the subsequent intestinal digestion stages, a steady improvement in particle size was observed in the colloidal solution digestates (FSPI-7.0 and FSPI-6.0), whereas the D [4,3] value of the curd digestates (FSPI-5.0 and FSPI-4.0) significantly declined. The D [4,3] value was 1.8–5.6-fold higher in the colloidal solution digestates than those in the curd digestates at I-30 and I-180. This result was consistent with the intuitive images, which showed finer particles in the curd digestates (FSPI-5.0 and FSPI-4.0) at I-30 and I-180, compared with the colloidal solution digestates.

##### Soluble Protein Content

Upon intestinal digestion, the FSPI-7.0 digestate at I-5 presented the highest soluble protein content (8.45 mg/mL) followed by FSPI-6.0 (5.79 mg/mL), whereas a lower soluble protein content was observed for FSPI-5.0 (5.10 mg/mL) and FSPI-4.0 (2.78 mg/mL). Subsequently, the colloidal solution digestates (FSPI-7.0 and FSPI-6.0) demonstrated a significant decline (*p <* 0.05) of soluble protein content and reached the lowest value at I-180. The soluble protein contents of the FSPI-4.0 curd digestates increased and peaked at I-30. This finding suggested that the colloidal solutions (FSPI-7.0 and FSPI-6.0) demonstrated a faster gastrointestinal transit rate than the curd samples (FSPI-5.0 and FSPI-4.0). FSPIs curd may possess a relatively slower physical mobility that postpones the degradation of soy protein [26].

##### Peptide Content

Peptide content was significantly higher in the intestinal digestates for all assayed samples, compared with that of the gastric digestates. A negligible increment in peptide content was observed in early intestinal digestion (I-5), compared with the initial stage of intestinal digestion (I-0), whereas a significant increment (1.8–2.4-fold) at I-30 was obtained for all of the investigated samples. The curd digestates possessed a higher peptide content at I-30 and a higher improvement at I-180 (2.6–2.9-fold). The values (4.76–5.96 mg/mL) were 2.6–3.3-fold higher (*p* < 0.05) than that of FSPI-7.0 (1.80 ± 0.44 mg/mL) and FSPI-6.0 (1.81 ± 0.04 mg/mL) at I-180. The results suggested that the peptide contents released from the curd digestates (FSPI-5.0 and FSPI-4.0) were higher and mainly observed at the medium and late digestion stages (I-30 and I-180).

### 2.5. Peptidomics Analysis of FSPI Digestates

#### 2.5.1. Peptidome Identification

**Figure 6:**
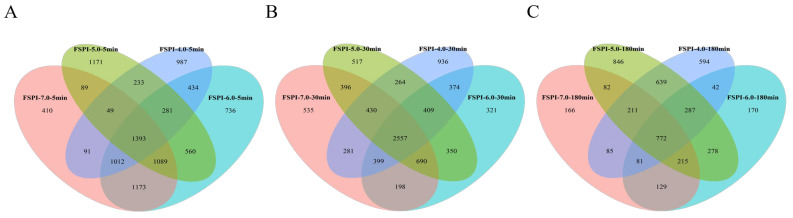
Venn diagram of the numbers of similar and unique peptides in the FSPI gastrointestinal digestates during dynamic intestinal digestion at (**A**) 5 min, (**B**) 30 min, and (**C**) 180 min.

Peptidomics is a useful tool for in-depth characterization of hydrolysis of protein in biological samples [27]. Figure 6 presents Venn diagrams based on the abundance of peptidome in SPIs and FSPIs intestinal digestates at 5 min (A), 30 min (B), and 180 min (C). Great differences existed in the peptidome obtained from four samples, especially between the digestates collected from the colloidal solutions and the curds. At the early intestinal digestion stage (I-5), the FSPI-6.0 digestate showed a peptidome abundance that was 1.3-, 1.4-, and 1.5-fold higher than those of FSPI-7.0, FSPI-5.0, and FSPI-4.0, respectively. The FSPI curd digestates (FSPI-5.0 and FSPI-4.0) elicited a comparatively lower rise in peptidome abundance at I-5, but showed a significant improvement in peptidome abundance at the medium digestion stage (I-30). The value (5613–5650 peptides) was relatively high (*p <* 0.05), compared with the value for the colloidal solution digestates (5298–5486 peptides). Subsequently, the abundance of peptidome in the colloidal solution digestates (FSPI-7.0 and FSPI-6.0) rapidly dropped at I-180, whereas the curd digestates (FSPI-5.0 and FSPI-4.0) showed a significantly slower decline. The values of 2711 and 3330 peptides obtained for the curd digestates were significantly high (*p <* 0.05), compared with the values for the colloidal solution digestates (1741–1974 peptides). A similar result was observed in polysaccharide–casein curd [28].

In addition, the four samples shared a proportion of identical peptides and showed a dynamic change. Along with the intestinal digestion, 1393, 2557, and 772 identical peptides were detected at I-5, I-30, and I-180, respectively. This finding indicated an increased trend in peptidome similarity between the four samples at early and medium stages (I-5 and I-30, respectively), then a decreased trend at the late stage (I-180). The colloidal solution digestates (FSPI-7.0 and FSPI-6.0) shared the greatest similarities at I-5 (4667 identical peptides), but all identical peptides shared between the colloidal solution digestates were significantly reduced at I-30 (3844 identical peptides) and I-180 (1147 identical peptides). The curd samples at I-5 shared a smaller portion of peptides (1956 identical peptides), whereas the quantity climbed to 3660 peptides at I-30, indicating a gradual increase in the similarity between the curd digestates. At I-180, the curd digestates (FSPI-5.0 and FSPI-4.0) shared relatively higher identical peptides (1909 identical peptides), compared with the colloidal solution digestates (1197 identical peptides). This result suggested that colloidal solutions possessed a similar digestive fate, mainly at the early stage (I-5), and the FSPI curd samples showed a similar digestive fate, mainly at the medium and late stages (I-30, I-180). This result was probably due to the hindrance of the gel network during early digestion [11].

#### 2.5.2. Soy Proteins Responsible for FSPI Digestate Peptidome

**Figure 7:**
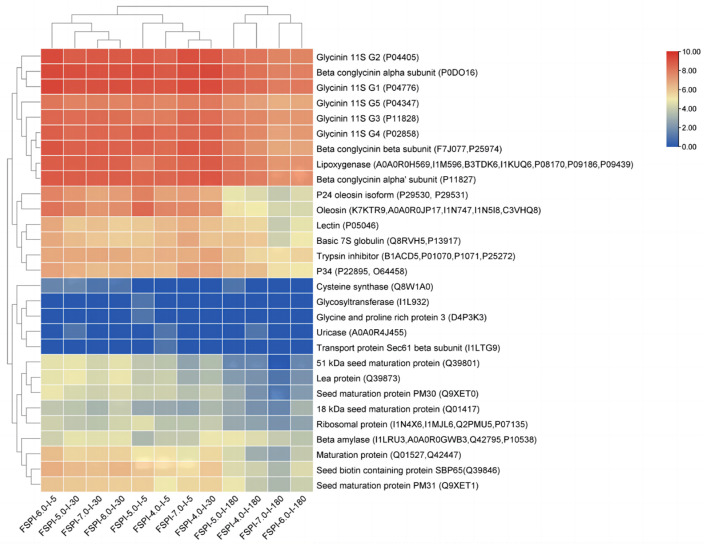
Clustering heat map representation of soy proteins responsible for the release of peptides identified in the FSPI digestates. I-5, I-30, and I-180 represent the gastrointestinal digestates collected from the artificial intestine at 5 min, 30 min, and 180 min, respectively.

Soy proteins responsible for the abundant peptidomes at different stages of the intestinal digestates were identified and shown by using a cluster heat map (Figure 7). In total, 29 proteins were identified. Glycinin and β-conglycinin were the top two sources of peptidome for all of the assayed samples. Compared with β-conglycinin, glycinin was a greater source of peptidome for all of the assayed samples, and this finding was considered to be related to the relatively lower accessibility of β-conglycinin to the digestive enzymes [29]. This result confirmed that the differences in colloidal solution/curd state caused by fermentation hardly alter the degradative preference between glycinin and β-conglycinin.

Glycinin was encoded by at least five genes (G1–G5). Glycinin G1–G5 were divided into groups I (G1–G3) and II (G4–G5), based on their sequence homology [30]. The quantity of peptides degraded from glycinin G1–G5 was in the order of G1 > G2 > G4 > G3 > G5 in all of the investigated samples, indicating that G1 and G2 were more easily digestible when compared with the other subunits. β-Conglycinin is a hetero-trimer composed of α, α′, and β subunits, which share an extensive sequence homology [31]. The α subunit was the preferred subunit to be degraded, and released the highest quantity of peptides, compared with the other two subunits. However, the quantity of peptides degraded from β-conglycinin α’ and β subunits varied between the colloidal solution samples and the curd samples. The preference of degradation was α′ subunit > β subunit in the FSPI curd samples (FSPI-5.0 and FSPI-4.0), whereas the colloidal solution samples (FSPI-7.0 and FSPI-6.0) exhibited the opposite order. This result may be due to the varied structural features and molecular arrangements of the α′ (72 kDa) and β subunits (52 kDa), which contributed to the different degradative priorities between the colloidal solution samples and the curd samples. A previous study indicated that the α subunit was more easily degraded than the other two subunits during in vitro digestion [14]. Our study agreed with this viewpoint and further confirmed that the LAB-induced gelation state of FSPI may change the degradative preference between the β-conglycinin α′ and β subunits.

In addition, FSPIs showed major discrepancies in the degradation of different subunits of glycinin and β-conglycinin. At the early intestinal digestion stage (I-5), FSPI-6.0 promoted the degradation from glycinin G2 and G3 and the β-conglycinin α and β subunits, demonstrating 1.1–1.6, 1.1–1.5 1.1–1.4 and 1.1–1.6-fold higher peptidome abundance than the other digestates. At the middle and late stages of intestinal digestion (I-30 and I-180, respectively), the curd samples (FSPI-5.0 and FSPI-4.0) demonstrated a major promotion in peptidome released from glycinin G2 and G4 and the β-conglycinin α and α’ subunits, which exhibited 1.1–1.5, 1.2–2.0, 1.1–1.6, and 1.1–1.8-fold higher peptidome abundance than the colloidal solution samples (FSPI-7.0 and FSPI-6.0), indicating a postponed but extensive hydrolysis. Between the FSPI curds, the FSPI-4.0 digestate rather than FSPI-5.0 at I-30 showed relatively abundant peptidome, and the hydrolysis was promoted in glycinin G4 and G5 and the β-conglycinin α subunits. The opposite result appeared at I-180.

In addition to glycinin and β-conglycinin, FSPIs with a lower fermentation-terminal pH (FSPI-6.0, FSPI-5.0, and FSPI-4.0), compared with that of FSPI-7.0, promoted peptide degradation from several proteins during intestinal digestion. These proteins, listed in the order of improvement in degradation degree, were lea protein, maturation protein, seed biotin containing protein SBP65, P24 oleosin isoform, oleosin, and ribosomal protein. Except for these proteins, lipoxygenase, P34, basic 7S globulin, trypsin inhibitor, lectin, and β amylase demonstrated dynamic changes in degradation patterns, similar to those of glycinin and β-conglycinin, showing a relatively higher degradation for FSPI-6.0 at I-5. Meanwhile, a significantly higher degradation was observed for the curd digestates (FSPI-5.0 and FSPI-4.0) at I-30 and I-180. The results confirmed that the FSPI curd samples demonstrated a postponed but extensive degradation of these proteins. P34 is known as Gly m Bd 30K and is a major allergenic protein [32]. Lectin and trypsin inhibitors are known as anti-nutritional factors [33]. Thus, their extensive degradation in the FSPI curd digestates can substantially improve the nutritional value of soy proteins.

The colloidal solution digestates (FSPI-7.0 and FSPI-6.0) showed an the absence of peptide degradation from several proteins throughout the intestinal digestion stage, compared with the curd digestates. These proteins included glycosyltransferase (I1L932), glycine and proline rich protein (D4P3K3), uricase (A0A0R4J455), and transport protein Sec61 β subunit (I1LTG9). I1L932 and D4P3K3 were detected only in the FSPI-5.0 digestate, whereas A0A0R4J455 and I1LTG9 were observed in the FSPI-4.0 digestate. These digestates were collected at I-5, indicating a rapidly degradative pattern.

#### 2.5.3. Predication of 3D Structure of Major Differential Protein Subunits

**Figure 8:**
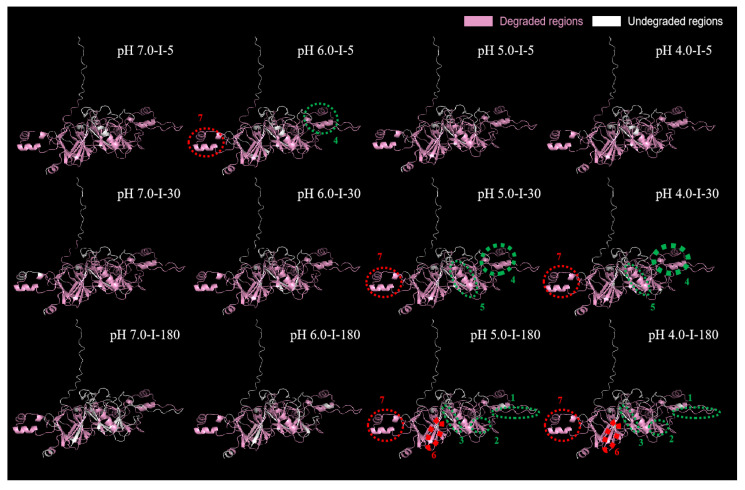
Distribution of gastrointestinal digestion sequence in the regions of glycinin G2 in the FSPI digestates. I-5, I-30, and I-180 represent the gastrointestinal digestates collected from the artificial intestine at 5 min, 30 min, and 180 min, respectively.

**Figure 9:**
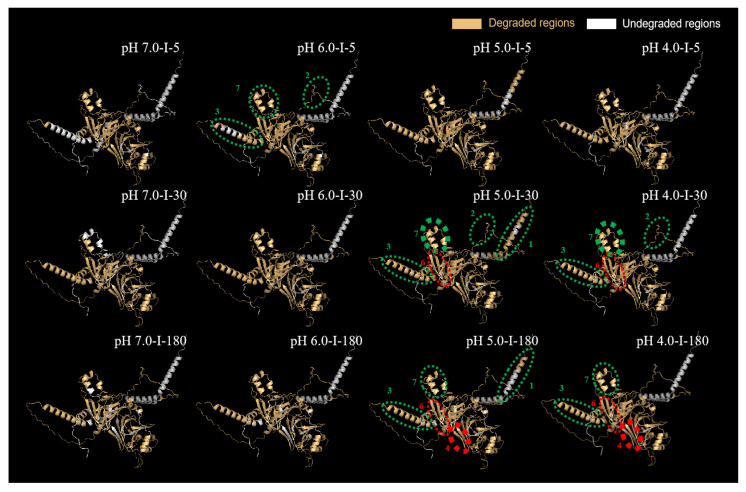
Distribution of gastrointestinal digestion sequence in the regions of the β-conglycinin α subunits in the FSPI digestates. I-5, I-30, and I-180 represent the gastrointestinal digestates collected from the artificial intestine at 5 min, 30 min, and 180 min, respectively.

Glycinin G2 and the β-conglycinin α subunit showed the greatest degradation discrepancies between samples, and they were further subjected to the 3D structural prediction. The results are shown in Figure 8 and Figure 9.

##### Glycinin G2

Glycinin G2 is composed of 485 amino acids, including signal peptide (M1–A18), acidic subunit (L19–K296), propeptide (R297–N300 and R481–A485), and basic subunit (G301–Q480). The variance in peptidome between the investigated samples was predominantly located in seven regions. They were further regarded as regions 1 (Q109–R117), 2 (A146–P156), 3 (A159–N166), 4 (F221–G250), and 5 (D276–Q283), which belonged to the acidic subunit (highlighted by green circles in Figure 8), and regions 6 (A349–K356) and 7 (A444–K465), which belonged to the basic subunit (highlighted by red circles in Figure 8).

In general, FSPIs obtained at low fermentation-terminal pH—FSPI-6.0, FSPI-5.0, and FSPI-4.0—elicited an overall potent degradation and demonstrated a dynamic promotion, shifting from exterior to interior regions at different digestive times. At I-5, the FSPI-6.0 digestate demonstrated a relatively high quantity of peptides released from regions 4 (F221–G250) and 7 (A444–K465) (highlighted by circles in Figure 8). Region 4 (F221–G250) was located at the acidic subunit, whereas region 7 (A444–K465) was located at the basic subunit. Both regions were located at the exterior of the 3D structure, showing a secondary structure of the α-helix. This finding indicated that the FSPI in the colloidal solution state facilitated the rapid hydrolysis of surface peptides, and this promotion had no preference for the acid/basic subunit. Along with the progress of intestinal digestion, the curd digestates (FSPI-5.0 and FSPI-4.0) at I-30 showed a major promotion at the acidic subunit, exhibiting a broader degradation coverage (84 % to 87%), compared with that of the colloidal solution digestates (83% to 86%). Region 4 (F221–G250) elicited the most potent promotion in the quantity of degrative peptides, with 1.2–1.4-fold higher values in the curd digestates than in the colloidal solutions (highlighted by bold circle in Figure 8). Of the two curd digestates, FSPI-4.0 showed a slightly higher quantity of degrative peptides than FSPI-5.0. In addition to those in region 4, the curd digestates had a relatively higher quantity of degrative peptides liberated from regions 7 and 5 in a descending order of the promotion degree (highlighted by circles in Figure 8).

The FSPI curd digestates (FSPI-5.0 and FSPI-4.0) at I-180 showed a major promotion at the basic subunit, with a broader degradation coverage of 86% to 87% compared with that of the colloidal solution (74% to 79%). Region 6 (A349–K356) elicited the most potent promotion in the degradation coverage by the curd digestates (100%), compared with that of the colloidal solution digestates (0%, no degradation). This region was located at the interior of the basic subunit (highlighted by bold circle in Figure 8). Of the two curd samples, the FSPI-5.0 digestate showed a higher degrative peptide quantity liberated from this region, compared with that of the FSPI-4.0 digestate. In addition, the curd digestates had a relatively higher quantity in degrative peptides at regions 3, 2, 1, and 7, in a descending order of the promotion degree (highlighted by circles in Figure 8). This result indicated that the FSPI curds elicited a slow but extensive hydrolysis pattern in the interior regions of glycinin, and this promotion occurred mainly at the basic subunit. A previous study suggested that the basic subunit of glycinin is more resistant to digestion than its acidic subunit [13]. The formation of curd by LAB may unfold the structure of glycinin, which will further lead to the exposure of cleavage sites, especially at the interior of the basic subunit, which facilitates the degradation of digestive proteases.

##### β-Conglycinin α Subunit

The β-conglycinin α subunit is composed of 605 amino acids, including signal peptides (M1–S22), propeptide (F23–K62), and the β-conglycinin α subunit chain (V63–Y605). The major variations between different FSPI digestates were observed in seven regions: regions 1 (M1–S22), 2 (P76–F106), 3 (Q161–H190), 4 (R202–G212), 5 (S388–R396), 6 (L491–T519), and 7 (E556–E575). Regions 1, 2, 3, 5, and 7 were located at the exterior of the 3D structure and demonstrated secondary structures consisting of α-helix and random coil (highlighted by green circles in Figure 9), whereas regions 4 and 6 were located at the interior of the 3D structure and demonstrated a β-sheet structure (highlighted by red circles in Figure 9).

The FSPIs at low fermentation-terminal pH (FSPI-6.0, FSPI-5.0, and FSPI-4.0) elicited an overall potent degradation and showed a dynamic promotion, shifting from the exterior to the interior regions at different digestive times. At I-5, the FSPI-6.0 digestate showed a relatively high quantity of degrative peptides, which were mainly derived from regions 2, 3, and 7 (highlighted by circles in Figure 9). These regions were located at the exterior of the 3D structure. However, along with the progress of intestinal digestion, the curd digestates (FSPI-5.0 and FSPI-4.0) at I-30 showed a major promotion at the exterior region 7 (E556–E575), showing a broader degradation coverage (100%) than that of the colloidal solution samples (34.1%) (highlighted by bold circle in Figure 9). Of the two curd samples, FSPI-5.0 digestate showed a higher degrative peptide quantity liberated from this region than that of the FSPI-4.0 digestate. In addition, the curd digestates had relatively higher peptide amounts at regions 1, 2, 3, and 6, in a descending order of the promotion degree (highlighted by circles in Figure 9).

The FSPI curd digestates (FSPI-5.0 and FSPI-4.0) at I-180 showed a major promotion at the interior region 4 (R202–G212), showing a broader degradation coverage (100%) compared with that of the colloidal solution (70%) (highlighted by bold circle in Figure 9). The FSPI-5.0 curd digestates again showed a greater quantity of degrative peptides from this region, compared with FSPI-4.0. In addition, the curd digestates had relatively higher peptide contents at regions 1, 3, 6, and 7, in a similar promotion degree (highlighted by circles in Figure 9). This finding indicated that the FSPI curds elicited a slow but extensive hydrolysis pattern in the interior region of β-conglycinin, and this promotion was mainly in the form of a β-sheet structure. A previous study suggested that β-conglycinin is more resistant to digestion than glycinin [29]. The formation of the curd structure may lead to the unfolding and rearrangement of the secondary structure, which will further increase the accessibility of digestive proteases to interior β-sheet areas.

## 3. Materials and Methods

### 3.1. Materials, Chemicals, SPIs and Microorganisms

Whole soybean seeds were obtained from a local supermarket in Nanjing, China, and stored at 4 °C until use. The molecular mass standard for mass spectrometry was purchased from Sangon Biotech (Shanghai, China). All chemical reagents used were of analytical grade and purchased from the Sigma-Aldrich Company (St. Louis, MO, USA).

Lactobacillus plantarum B1-6 was isolated from Xingjiang Kirgiz boza, which is a traditional cereal-based drink derived from the Xinjiang Vygur autonomous region of China. The strain has the gene accession number KM200717.

### 3.2. Preparation of SPIs and FSPIs

#### 3.2.1. SPI Preparation

SPI extraction was conducted in accordance with a previous “standard procedure” [34].

#### 3.2.2. Inoculum Preparation

*L. plantarum* B1-6 strain was activated for two successive transfers in de Man, Rogosa, and Sharpe broth (MRS, pH 6.2) at 37 °C. The bacteria were harvested by centrifugation at 7500× *g* at 4 °C for 10 min and washed twice with sterile physiological saline, then resuspended in an equal volume of sterile physiological saline to prepare the bacterial suspension for inoculation [35].

#### 3.2.3. FSPI Preparation

SPI was dissolved in water at 4.0% *w*/*v* to prepare the SPI solution. The pH was adjusted to 7.0 with HCl (1 mol/L). The SPI solution was heated at 85 °C for 10 min to decrease the endogenous microbial content. Sterilized glucose (0.5 g/mL) was added to the solution at a final concentration of 2% *w*/*v*. Fermentation was initiated by the inoculation of *L. plantarum* B1-6 at a final concentration of 3.0% *v*/*v*. A pH meter (Testr30, Oakton, Singapore) was applied to constantly monitor the pH of the SPI and FSPI. Fermentation was terminated as the pH dropped to 7.0, 6.0, 5.0, and 4.0. Fermentation was conducted in triplicate.

### 3.3. Protein Gelation

#### 3.3.1. Microbiological Analysis

SPI fermentation was monitored by the determination of the number of viable cells of *L. plantarum* B1-6 after growth on the MRS agar plate (Oxoid-CM0361, Unipath, Basingstoke, UK) at 37 °C for 48 h. Cell counts were performed in three replicates and expressed as log CFU mL^−1^.

#### 3.3.2. Cryo-electron Microscopy (Cryo-EM)

Microstructures of FSPI terminated at varied pH levels were monitored by Cryo-EM. Scanning of sample microstructure in situ was conducted using an instrument (Hitachi SU8010, Tokyo, Japan) with a cryofixation apparatus at 3.0 kV. The temperature was decreased to that of liquid nitrogen (−196 °C) or below, using an ultra-rapid cooling technique. The SPI and FSPI samples were cryo-fractured under vacuum to expose their internal microstructures. Then, the cryo-fractured SPI and FSPI samples were observed with a scanning electron microscope (Quanta 650, FEI, Hillsboro, OR, USA).

#### 3.3.3. Interactions between Soy Proteins

Interactions between soy proteins were investigated in accordance with a previous method, with some modifications [36]. To determine the interaction forces between soy proteins—ionic bond, hydrogen bond, hydrophobic forces, and disulfide bond—we solubilized SPIs and FSPIs in solutions A (0.6 mol/L of NaCl), B (0.6 mol/L of NaCl with 1.5 mol/L of urea), C (0.6 mol/L of NaCl with 8 mol/L of urea), and D (0.6 mol/L of NaCl with 8 mol/L of urea and β-metcaptoethanol at pH 7.0). Several extraction steps were carried out under identical conditions, including homogenization (at 5000 r/min for 2 min), extraction (at 4 °C for 1 h), and centrifugation (at 10,000× *g* for 20 min). This step is referred to as “extraction” in the following text.

A total of 5 mg lyophilized samples (SPIs and FSPIs) was mixed thoroughly with 1 mL of solution A, and the supernatant (S1) was obtained by the first extraction. The sediment was subsequently added to 1 mL of solution B, and the supernatant (S2) was collected by performing the second extraction. The sediment was added to 1 mL of solution C, and the supernatant was collected as S3 after the third extraction. The sediment was added to 1 mL of solution D, and the supernatant was collected as S4 after the fourth extraction. The soluble protein contents of S1, S2, S3, and S4 were analyzed using the Bradford method [19]. This analysis was performed in triplicate.

### 3.4. In Vitro Dynamic Gastrointestinal Digestion

In vitro dynamic gastrointestinal digestion was performed by a mechanized soft rat model (Bionic Rat Model II+, Xiao Dong Pro-health Instrumentation Co Ltd., Suzhou, China). Enzyme cocktails during in vitro dynamic gastrointestinal digestion were consistent with those of a previous study [37]. Simulated gastric fluid (SGF) was prepared by dissolving pepsin (0.27 g/L) and mucin (1.5 g/L) in SGF, which was composed of NaHCO_3_ (25 mmol/L), KCl (6.9 mmol/L), NaCl (47.2 mmol/L), KH_2_PO_4_ (0.9 mmol/L), MgCl_2_(H_2_O)_6_ (0.1 mmol/L), and (NH_4_)_2_CO_3_ (0.5 mmol/L), and the pH was adjusted to 1.6. Simulated intestinal fluid (SIF) was obtained by dissolving pancreatin (5.62 g/L) and bile salts (8.17 g/L) in SIF, which was composed of KCl (6.8 mmol/ L), NaCl (38.4 mmol/L), KH_2_PO_4_ (0.8 mmol/L), MgCl_2_(H_2_O)_6_ (0.33 mmol/L), and NaHCO_3_ (85 mmol/L), and the pH was adjusted to 7.0. Before digestion, 0.6 mL SGF was pre-added to SPI and FSPI samples in an artificial rat stomach to mimic the real in vivo digested condition. Gastric and intestinal digestions were conducted for 120 min and 180 min, respectively. The temperature of gastrointestinal digestion was set at 37 °C. Artificial gastric juice was constantly provided by a syringe pump, and the injection rate was set at 52 µL min^−1^. The movement of the rat stomach in vivo was mimicked by pressing the silicon stomach model at a rate of three compressions and 12 extrusions per minute. By passing through an artificial pylorus, the gastric digestates reached the duodenal section, where artificial intestinal fluid was constantly provided by a syringe pump, and the injection rate was set at 52 µL min^−1^. The artificial intestinal digestion was carried out using six sets of rolling extrusion plates to mimic the movement in vivo. The rate of rolling extrusion was set at 12 extrusions per minute. Samples of SPI and FSPI digestates were obtained during gastric digestion at 5 min, 30 min, and 120 min (G-5, G-30, and G-120, respectively) and intestinal digestion was obtained at 5 min, 30 min, and 180 min (I-5, I-30, and I-180, respectively). All collected samples were instantly subjected to boiling temperature for 5 min. The pH was evaluated by a pH meter (Testr30, Oakton, Singapore). The gastrointestinal digestions were performed in triplicate.

### 3.5. Particle Size Distribution

Particle size distribution was measured by an integrated laser light scattering (ILLS) instrument (Mastersizer 3000, Malvern Southborough, MA, USA). The refractive index used for the scatterers (FSPI digestates) was set as 1.46, and the refractive index of the water dispersant was 1.33. The size distribution was characterized by D [4,3] and D [v,0.90]. D [4,3] was defined as the volume-weighted mean diameter and represented the size of sample particles. The analysis was performed in triplicate.

### 3.6. Soluble Protein Content

Soluble protein was tested by the Bradford assay [19]. Bovine serum albumin was provided as a standard, according to the method. Three replicates were performed.

### 3.7. Peptide Content Measurement

Peptide content was tested by O-phthaldialdehyde (OPA) assay. The OPA solution was prepared by following a previous method [23]. Crude peptides were extracted by the addition of trichloroacetic acid (TCA) reagent and centrifugation at 12,000× *g* for 15 min to remove insoluble proteins. A total of 2 mL of reaction reagent was applied to 0.5 mL of crude peptide solution and incubated for 2 min at room temperature. The absorbance was observed by a spectrophotometer (U-4100, Hitachi Ltd., Tokyo, Japan) at 340 nm. Triplicate determinations were conducted.

### 3.8. Peptide Identification

Liquid chromatography with tandem mass spectrometry (Thermo Fisher Scientific, Palo Alto, CA, USA) was applied to the identified peptides, in accordance with a previously described protocol [16]. The peptides of SPI and digestates were extracted by the addition of the TCA reagent and centrifugation at 12,000× *g* for 15 min. Desalination of the digestates was performed using a MonoSpin C18 desalination column. The mobile phase A solution (98% water, 2% acetonitrile, 0.1% fomic acid), B solution (98% acetonitrile, 2% water, 0.1% fomic acid), pre-column (300 µm × 0.5 mm, 3 µm), and analytical column (3 µm, 75 µm × 150 mm, Welch Materials, Inc.) were prepared at the spray voltage of 1.9 KV. The peptides separated by liquid phase were ionized by a nano-electrospray ionization source and entered into a tandem mass spectrometer Q-Exactive HFX (Thermo Fisher Scientific, San Jose, CA, USA) for detection, and raw data for mass detection (.raw) were generated. Raw mass spectrometry files were retrieved and analyzed by MaxQuant (version 1.6.2.6) and searched against the Uniprot reference database for Glycine max. The predication of a three-dimensional (3D) structure of glycinin G2 and the β-conglycinin α subunit was investigated using a Uniprot spatial model (PDB ID: AF-O15488 and P0DO16-F1-model).

### 3.9. Statistical Analysis

One-way analysis of variance and Duncan’s multiple comparison tests were used to determine significant differences among means (*p* < 0.05) by using Statistical Product and Service Solutions (SPSS) software version 17.0 (SPSS Inc., Chicago, IL, USA). The clustering heat map was used to show soy proteins responsible for the release of peptides. The graphs were generated using the Heatmapper (http://www.heatmapper.ca (accessed on 4 March 2022)).

## 4. Conclusions

The LAB fermentation-induced soy protein matrix shifted from colloidal solutions to curds at approximately pH 5.5. The curd samples (FSPI-5.0/4.0), compared with the colloidal solution samples (FSPI-7.0/6.0), demonstrated a microstructure of a denser gel network structure and a major shift of dominant interaction from ionic bond to hydrophobic force and disulfide bond. The results from dynamic gastrointestinal digestion demonstrated that the FSPI curd samples possessed a relatively slow physical mobility and showed a postponed degradation but an extensive proteolysis pattern at medium and late gastrointestinal digestion stages. Compared with the colloidal solution samples, the curd samples exhibited a major promotion in the degradation of glycinin and β-conglycinin, in which glycinin G2 and the β-conglycinin α subunit were the predominant subunits to be promoted at medium and late gastrointestinal digestion stages. The difference was located mainly at the exterior of the 3D structure at I-30, namely, glycinin G2 acidic subunit in region 4 (F221–G250) and β-conglycinin α subunit in region 7 (E556–E575), and shifted to the interior of the 3D structure at I-180, namely, glycinin G2 basic subunit in region 6 (A349–K356) and β-conglycinin α subunit in region 4 (R202–G212).

This study confirmed that LAB fermentation facilitated the degradation of soy proteins in gastrointestinal digestion, especially when curds were formed. The slow but extensive hydrolysis of FSPI curd samples indicated that LAB fermentation led to the exposure of interior cleavage sites at the medium and late stages of gastrointestinal digestion. These findings match the previously investigated results reported for cow milk yogurt [38]. This study provides insights into the gastrointestinal digestive pattern of FSPI and valuable information and theoretical bases for understanding the nutritional values of soy-based yogurt.

## Data Availability

The data presented in this study are available on request from the corresponding author.

## Sample Availability

Samples are available from the authors.
